# Past and present: conditions of life during childhood and mortality of older adults

**DOI:** 10.1590/S0034-8910.2015049005555

**Published:** 2016-01-12

**Authors:** Marília Miranda Forte Gomes, Cássio Maldonado Turra, Moema Gonçalves Bueno Fígoli, Yeda A O Duarte, Maria Lúcia Lebrão

**Affiliations:** I Faculdade do Gama. Universidade de Brasília. Brasília, DF, Brasil; II Centro de Desenvolvimento e Planejamento Regional. Faculdade de Ciências Econômicas. Universidade Federal de Minas Gerais. Belo Horizonte, MG, Brasil; III Departamento de Enfermagem Médico-Cirúrgica. Escola de Enfermagem. Universidade de São Paulo. São Paulo, SP, Brasil; IV Departamento de Epidemiologia. Faculdade de Saúde Pública. Universidade de São Paulo. São Paulo, SP, Brasil

**Keywords:** Aged, Mortality, Child Development, Social Conditions, Socioeconomic Factors

## Abstract

**OBJECTIVE:**

To analyze whether socioeconomic and health conditions during childhood are associated with mortality during old age.

**METHODS:**

Data were extracted from the SABE Study (*Saúde, Bem-estar e Envelhecimento* – Health, Welfare and Aging), which were performed in 2000 and 2006. The sample consisted of 2004 (1,355 living and 649 dead) older adults. The statistical analysis was performed based on Poisson regression models, taking into account the time variation of risk observed. Older adults’ demographic characteristics and life conditions were evaluated, as were the socioeconomic and lifestyle conditions they acquired during their adult life.

**RESULTS:**

Only the area of residence during childhood (rural or urban) remained as a factor associated with mortality at advanced ages. However, this association lost significance when the variables acquired during adulthood were added to the model.

**CONCLUSIONS:**

Despite the information regarding the conditions during childhood being limited and perhaps not accurately measure the socioeconomic status and health in the first years of life, the findings of this study suggest that improving the environmental conditions of children and creating opportunities during early adulthood may contribute to greater survival rates for those of more advanced years.

## INTRODUCTION

The term ‘life conditions during childhood or early ages’ is widely used in literature^22^ and refers to a heterogeneous set of factors made up of the nutritional status from the womb to childhood, physiological growth and development in the early years of life, exposure and contraction of infectious and parasitic diseases, contact with stressful environments and, more generally, through experiences associated with the family’s socioeconomic conditions during childhood.

Investigations were performed, beginning from the late decades of the 20^th^ century, whose purpose was to study whether individuals exposed to socioeconomic conditions and adverse health as children are more or less likely to survive through adulthood or through to more advanced ages. According to Preston, Hill and Drevenstedt,^25^ at least four mechanisms related to conditions during childhood as well as adult mortality exist. These mechanisms can work directly, representing the physiological influence of health conditions during childhood and adult mortality and, indirectly, when associated with non-physiological variables.

When working directly, these mechanisms could produce a positive or negative association regarding mortality risks during adulthood. For the positive association, – scarring or side effects – adverse conditions and diseases acquired during childhood may reduce the probability of survival at more advanced ages. Low birth weight and malnourishment during the first years of life can result in physiological damage. In addition to harming the healthy and full development of functional organs, malnutrition during childhood can lead to individuals being more vulnerable to non-communicable diseases, especially cardiovascular diseases and diabetes, which may contribute to an increased risk of death.^1^ ^,^ ^6^ ^,^ ^7^ ^,^ ^10^

The negative relationship is associated to immunity that is built up by the individual throughout his/her life. The living individuals, who were often exposed to childhood diseases, such as flu, asthma and smallpox, may be at lesser risk of death during adulthood than those who had enjoyed healthier environments. This successive exposure to illnesses may strengthen the autoimmune system and reduce the risk of death.^9^ ^,^ ^22^

As regards the direction of the indirect relationship, the mechanisms that have positive associations can be correlated to the conditions of the environment in which the individuals live. Individuals born in favorable circumstances (father or mother belonging to higher status occupational classes, healthy available food, housing in areas that are less exposed to epidemics and that have more access to education and quality health services) tend to retain these benefits throughout life and are more likely to live longer throughout adulthood and old age. The positive indirect relationship indicates that individuals who have experienced adverse conditions related to their environment, during childhood, tend to have a higher risk of mortality during their adult years.^9^ ^,^ ^23^ ^,^ ^27^ On the other hand, the negative associations are related to the selective nature of mortality, i.e., regardless of the conditions, weaker individuals die at a young age while only the strongest survive to more advanced years.^25^ ^,^ ^27^

Most of Brazil’s older adults population had a childhood characterized by precarious social and economic conditions and health. If the situation of the urban and social structure of cities in the late 19^th^ and early 20^th^ centuries was poverty and poor living conditions, then today, cities are urbanized and offer a greater range of services and information that contribute to better health and lifestyle.^21^ The average life expectancy of the world’s population, more specifically that in Brazil, grew significantly during the 20^th^ century. Studies such as these^13^ ^,^ ^20^ ^,^ ^24^ ^,^ ^25^ are increasingly important to understand the factors that are associated with mortality in different segments of the population.^9^ Performing research on the determinants associated with childhood conditions and survival during later years contribute not only to topics such as adult mortality and public policies, which are focused on the older population, but also to plan and implement social policies that are focused on the early years of life. This can ensure greater survival rates for individuals during adulthood.^27^

Thus, the objective of this article is to analyze whether socioeconomic and health conditions during childhood are associated with mortality during old age.

## METHODS

This article used data from a Brazilian study known as *SABE* – *Saúde, Bem-estar e Envelhecimento *(Health, Welfare and Aging), which was performed in 2000 and 2006 in the city of Sao Paulo and was based on a representative sample of the urban population aged 60 years or over.^26^ The SABE sample was built from two different phases. 90 households in 72 census sectors were visited during the first phase using the sampling by conglomerates method. A permanent record, available at the Department of Epidemiology at the *Faculdade de Saúde Pública of the Universidade de São Paulo*, was extracted from the *Pesquisa Nacional por Amostra de Domicílios *(PNAD – National Household Sample Survey) of the Brazilian Institute of Geography and Statistics (IBGE). Eligible individuals (older adults aged 60 years or more, non-institutionalized and residents in urban areas of the city of Sao Paulo) were identified and invited to participate in the interviews. During the first phase of the research,^16^ ^,^ ^17^ 1,568 older adults were interviewed. During the second phase, the sample was increased by 575 older adults aged 75 years or more, these new additions were living in houses close to the selected sectors or, at most, within the boundaries of the districts to which the drawn sectors belonged, thereby totaling 2,143 older adults. The objective of this was to compensate for the effect of mortality and reach the desired number of interviews for this age group.

Among the 2,143, non-institutionalized older adults (100%) living in urban areas of the city of Sao Paulo, who were participants from the first phase of the study in 2000, 1,115 (52.0%) were interviewed again between July 2006 and December 2007. The difference between the number of respondents in 2000 and 2006 (1,028) was due to deaths of numerous causes (30.0%), institutionalizations (1.0%), moving house (2.0%), not found (7.0%) and refusals to participate (8.0%). The survival condition of each older adults was determined at the end of the observation period (Figure). The analyzed sample was 2,004 older adults, of which 1,355 were survivors and 649 deceased. The difference of 139 excluded individuals was composed by those who were interviewed in 2000, but for whom it was possible to determine their survival condition in 2006. This exclusion did not alter the distribution pattern of the variables used in the analysis, based on the results of the Chi-square tests (p > 0.10).

**Figure:**
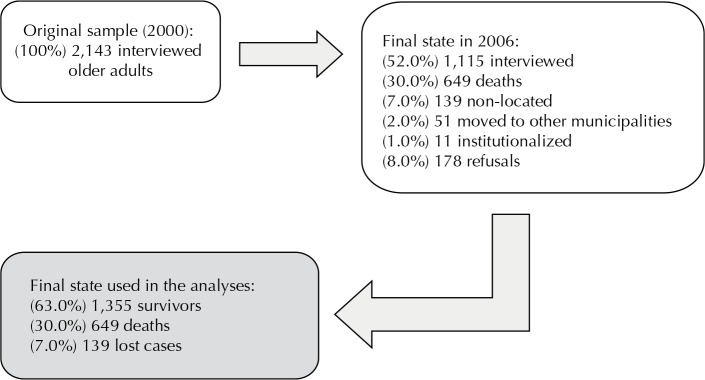
Final state considered in the analyzed sample. Sao Paulo, SP, Southeastern Brazil, 2000 to 2006.

Source: 2000-2006 SABE study.

We selected 15 variables to analyze the relationship between mortality and conditions in the early years of life. The variables were organized into four groups (Table 1): basic demographic characteristics; conditions during early life; social and economic conditions acquired during adulthood; and lifestyle during adulthood. The explanatory variables refer to information declared in 2000, the year of the first interview of the SABE study (baseline).

**Table 1:** Explanatory variables used to analyze the relationship between mortality and conditions in the early years of life. Sao Paulo, SP, Southeastern Brazil, 2000 to 2006.

Variable	Description*	Categorization
Nationality	Were you born in Brazil? (A2)	Yes
		No
Age groups	Year of birth (A1a)	60 to 69 years (sexagenarian)
	Year of death	70 to 79 years (septuagenarian)
	Year of the first interview (int_ANO1)	≥ 80 years
	Year of the second interview (int_ANO2)	
Gender	Gender of respondent (C18)	Man
		Woman
Starvation	During the first 15 years of your life, has there ever been a time in which you did not have enough to eat or go hungry? (C30)	Yes
		No
Economic situation of the family	How would you describe your family’s economic situation during the most part of your first 15 years life? (C26)	Good
		Average/Bad
Experience living in the countryside	During the first 15 years of your life, have you ever spent 5 years or more living in the countryside? (A4b)	Yes
		No
Self-assessment of health	For the majority of your early 15 years of life, how you would describe your health at that time? (C27)	Excellent/Good
		Bad
Bedridden	For the first 15 years of your life, have you ever spent one month or more in bed due to some health problems? (C29)	Yes
		No
Education level	Number of years spent studying (A5a, A5b, A6)	None
		1 to 7 years
		≥ 8 years
Do you have your own house?	This house belongs to: (J2)	Yes
		No
Nutritional status	How would you consider your nutritional status? (C22i)	Well nourished
		Under nourished
Marital status	Conjugal status (A13a, A15, A16)	Married
		Divorced/Separated
		Widow
		Single
Smoking habit	Do you smoke? (C24)	Yes
		I used to, but I do not anymore
		I have never smoked
Alcohol use	Over last three months, on average, how many days a week did you drink alcohol? (C23)	Did not drink alcohol
	Over the last three months, on days when you drank alcohol, how many glasses did you have per day? (C23a)	Did drink alcohol
Physical activity	Over the last 12 months, have you regularly, 3 times a week, done any exercise or vigorous physical activities? (C25a)	Yes
		No

Source: Own production.

* The codes in parentheses in the description of the variables refer to the identification of the variable in the database from the SABE study.

One hundred and forty-eight of the 2,004 analyzed older adults had not answered some of the explanatory variables. Chi-square tests were performed to examine the randomness of these missing answers and the possible loss of statistical significance in estimating the multivariate models. Excluding these missing values did not alter the distribution pattern of the variables. Thus, the final sample was composed of 1,856 older adults, 1,276 of which were alive and 580 had deceased.

Poisson regression models were used to analyze the relationship between the number of deaths from 2000 to 2006 and the selected explanatory variables.^15^ ^,^ ^a^ The relationship between each explanatory variable and response variable, represented by the condition of the individuals (living, or deceased), were separately investigated. Explanatory variables that had a p < 0.20 were deemed eligible for composing the multiple Poisson regression models. This procedure was adopted so as to make the choice regarding the explanatory variables, based on international literature, more economical.^14^ ^,^ ^23^ The variables excluded from the initial list referred to the conditions at the beginning of life: economic situation of the family, self-assessment of health and having stayed in bed for one month or more due to health problems. Four models were estimated to examine the effects of childhood conditions on mortality.

According to Garcia et al,^11^ detecting a possible presence of multicollinearity (perfect or approximate linear dependence between at least two explanatory variables)^11^ ^,^ ^12^ requires analyzing the Variance Inflation Factor (VIF) in each of the models. The VIF is indicative of multicollinearity problems when values greater than 10 are present. The mean value of this measurement was not greater than 1.5 and no variable presented a VIF greater than 2.7 in any of the estimated multivariate models.

The sampling weights and delineated sampling plan for the SABE study were considered for the estimates of the models.^26^ For this purpose, specific routines, available in the Stata software, version 9.0, were used for processing the data from the complex samples.^12^ A more detailed description of how the sampling weights were calculated can be found in Silva.^26^

## RESULTS

The level of mortality was significantly higher for older male adults and for those in more advanced age groups. The survival rate was higher when the individuals had a higher education level and socioeconomic status (being a homeowner) (Table 2). A statistically higher mortality level was observed for older adults who described their nutritional status as bad, as it was for widows, smokers, those who did not exercise regularly and those who regularly drank alcohol. The level of mortality was 26.6% lower for older adults who had lived at least five years in an urban area until the age of 15 than for those who had lived in rural areas.

**Table 2:** Relative distribution and univariate analysis, by living condition, according to explanatory variables selected for the multiple models. Sao Paulo, SP, Southeastern Brazil, 2000 and 2006. (N = 1,856)

Variable	Relative frequency		Univariate analysis		
	Living	Dead	Coefficient	MRR	p
Nationality					
Brazilian	90.6	85.8	–		
Foreign	9.4	14.2	0.293	1.340	0.120
Age groups					
Sexagenarian	45.1	20.3	–		
Septuagenarian	39.3	36.9	0.558	1.747	< 0.001
≥ 80 years	15.7	42.7	1.415	4.116	< 0.001
Gender					
Man	37.1	52.0	–		
Woman	62.9	48.0	-0.451	0.637	< 0.001
Starvation					
Yes	19.7	16.1	–		
No	80.3	83.9	0.176	1.192	0.131
Economic situation of the family					
Good	30.7	32.0	–		
Average/Bad	69.3	68.0	-0.766	0.465	0.502
Experience living in the countryside					
Yes	60.8	70.1	–		
No	39.2	29.9	-0.310	0.734	0.018
Self-assessment of health					
Excellent/Very good/Good	93.7	96.1	–		
Average/Bad	6.3	3.9	-0.283	0.753	0.260
Bedridden					
Yes	7.8	7.7	–		
No	92.2	92.3	-0.007	0.993	0.970
Education level					
None	20.0	33.0	–		
1 to 7 years	63.4	56.2	-0.465	0.628	< 0.001
≥ 8 years	16.6	10.8	-0.793	0.453	< 0.001
Do you have your own house?					
Yes	83.1	77.8	–		
No	16.9	22.2	0.345	1.412	0.008
Nutritional status					
Well nourished	94.4	91.8	–		
Under nourished	5.6	8.2	0.332	1.394	0.044
Marital status					
Married	56.0	50.1	–		
Divorced/Separated	7.6	4.8	-0.121	0.886	0.603
Widow	32.1	40.9	0.273	1.314	0.039
Single	4.3	4.3	0.187	1.206	0.371
Continue					
Continuation					
Smoking habit					
Yes	11.8	16.9	–		
Have smoked, but not anymore	30.5	35.6	-0.379	0.685	0.010
Never smoked	57.8	47.5	-0.655	0.520	< 0.001
Alcohol use					
Did not drink alcohol	67.7	76.2	–		
Did drink alcohol	32.3	23.8	-0.298	0.743	0.031
Physical activity					
Yes	29.2	12.6	–		
No	70.8	87.4	0.814	2.256	< 0.001

Source: 2000-2006 SABE study.

MRR: Mortality Rate Ratio

Model 1, multiple, included information that represents the conditions during early life and the basic demographic characteristics. The variables ‘nationality’ (p = 0.978) and ‘starvation during childhood’ (p = 0.302) were not associated with mortality. Older adults who had lived in urban areas during the early years of their lives showed death rates that were approximately 20.0% lower than those who lived in rural areas (p < 0.10) (Table 3).

**Table 3:** Results of the multivariate models estimated for analyzing the relationship between conditions during childhood and mortality in older adults. Sao Paulo, SP, 2000 and 2006.

Variable	Model 1			Model 2			Model 3			Model 4		
	Coefficient	MRR	p	Coefficient	MRR	p	Coefficient	MRR	p	Coefficient	MRR	p
Individual characteristics												
Nationality												
Brazilian	–	1.000		–	1.000		–	1.000		–	1.000	
Foreign	0.005	1.005	0.978	0.059	1.060	0.751	0.133	1.142	0.481	0.136	1.146	0.459
Age groups												
Sexagenarian	–	1.000		–	1.000		–	1.000		–	1.000	
Septuagenarian	0.576	1.779	< 0.001^a^	0.527	1.694	0.001^a^	0.551	1.735	< 0.001^a^	0.564	1.757	< 0.001^a^
≥ 80 years	1.468	4.338	< 0.001^a^	1.346	3.842	< 0.001^a^	1.330	3.781	< 0.001^a^	1.347	3.845	< 0.001^a^
Gender												
Man	–	1.000		–	1.000		–	1.000		–	1.000	
Woman	-0.535	0.586	< 0.001^a^	-0.680	0.507	< 0.001^a^	-0.641	0.527	< 0.001^a^	-0.640	0.528	< 0.001^a^
Conditions during early life												
Starvation												
Yes	–	1.000		–	1.000		–	1.000				
No	0.127	1.135	0.302	0.194	1.215	0.134	0.220	1.246	0.110			
Experience living in the countryside												
Yes	–	1.000		–	1.000		–	1.000				
No	-0.226	0.798	0.066^b^	-0.131	0.877	0.274	-0.097	0.908	0.414			
Adulthood												
Education level												
None				–	1.000		–	1.000		–	1.000	
1 to 7 years				-0.254	0.775	0.015^c^	-0.135	0.874	0.195	-0.151	0.860	0.129
≥ 8 years				-0.534	0.586	0.012^c^	-0.347	0.707	0.100	-0.366	0.693	0.082^b^
Do you have your own house?												
Yes				–	1.000		–	1.000		–	1.000	
No				0.331	1.392	0.009^a^	0.299	1.349	0.014^c^	0.290	1.336	0.019^c^
Nutritional status												
Well nourished				–	1.000		–	1.000		–	1.000	
Under nourished				0.397	1.488	0.018^c^	0.233	1.263	0.134	0.193	1.212	0.221
Marital status												
Married				–	1.000		–	1.000		–	1.000	
Divorced/Separated				0.033	1.034	0.888	-0.001	0.999	0.997	-0.013	0.987	0.949
Widow				0.175	1.191	0.196	0.167	1.182	0.216	0.158	1.172	0.237
Single				0.255	1.290	0.202	0.251	1.285	0.162	0.226	1.253	0.191
Smoking habit												
Yes							–	1.000		–	1.000	
Have smoked, but not anymore							-0.469	0.626	0.002^a^	-0.484	0.616	0.000^a^
Never smoked							-0.634	0.531	< 0.001^a^	-0.636	0.529	< 0.001^a^
Alcohol use												
Did not drink alcohol							–	1.000		–	1.000	
Did drink alcohol							-0.276	0.759	0.035^c^	-0.273	0.761	0.035^c^
Physical activity												
Yes							–	1.000		–	1.000	
No							0.611	1.842	< 0.001^a^	0.609	1.838	< 0.001^a^
Sample size	1.856			1.856								
Constant	-3.56		< 0.001^a^	-3.47		< 0.001^a^	-3.54		< 0.001^a^	-3.39		< 0.001^a^
F value	26.81			23.02								
p-value	< 0.001			< 0.001								

Note: The first model includes the basic demographic characteristics of individuals and the variables that represent their living conditions during childhood. Models 2 and 3 were intended to measure the role of the intervening variables that represent the characteristics acquired during adulthood: model 2 includes the social and economic conditions and model 3 incorporates the life style variables. Model 4 was estimated to exclude the variables regarding the conditions during early life. Its objective is to examine the extent to which omitting the conditions during childhood affects the explanatory power of traditional adult mortality models, which only take the characteristics acquired during the later stages of life into account.

Source: 2000-2006 SABE study.

MRR: Mortality Rate Ratio

^a^ p < 0.01.

^b ^p < 0.10.

^c^ p < 0.05.

The variable ‘experience living in the countryside’ lost its statistical significance (p > 0.10) in model 2, which included the socioeconomic conditions acquired during adulthood. Thus, older adults who had higher education levels, who owned their own home or who positively evaluated their nutritional status had a higher survival rate, even in the presence of the controls to the conditions during childhood (p < 0.05). Model 3 included variables regarding the lifestyle of older adults. Variables concerned with conditions during childhood still had no statistical significance (p > 0.10). Educational level effects became marginally significant (p = 0.10) while the nutritional state stopped being significant (p > 0.10).

In model 4, when the variables that represent conditions at the beginning of life were excluded, the effects that socioeconomic status and lifestyle had on mortality showed almost no change (all significant at the 5% level). Education, which in model 3 was marginally significant at a 10.0% level, went to p = 0.082 (Table 3).

## DISCUSSION

When only considering the demographic variables and conditions during childhood, a significant negative relationship existed between mortality and not having lived in rural area until 15 years of age (p < 0.10) observed during the first multiple analysis, namely, older adults who had lived in urban areas as children showed a lower prevalence of deaths than those who had lived in the countryside. However, after the socioeconomic conditions and current lifestyle were separately controlled, the effect of having lived in rural towns during the early years of life on the risk of mortality for older adults ceased to be statistically significant. When the variables that represent the conditions at the beginning of life were excluded from the multiple model, the effects of the socioeconomic and lifestyle variables on mortality showed almost no change.

The results of this study confirm those performed in other countries. Conditions during childhood cease to be statistically significant when the current socioeconomic status and lifestyle are added to the model, which is in line with a report by: Bobak et al,^3^ in study on the determinants of adult and older adult mortality in a population residing in Russia; by Hayward and Gorman,^13^ for an American older adults population; by Beebe-Dimmer et al,^2^ among adult women and older adult residents in California; and by Yi, Gu and Land,^27^ while examining the older adults population residing in China (p < 0.10). The authors conclude that conditions acquired during adult life significantly act as intermediary factors in the relationship between socioeconomic characteristics and health care during the first years of life and mortality at more advanced ages.

Results in the literature are contentious with regards to older adults who had lived in rural areas as children presenting higher mortality rates. Contrary to what was observed by Hayward and Gorman^13^ and Preston, Hill and Drevenstedt,^25^ during this study and that by Yi, Gu and Land,^27^ older adults who had lived in urban areas as children showed a lower risk of mortality compared to those who live in rural locations. According to Preston et al,^25^ this may be explained by the fact that these urban areas had better socioeconomic, environmental conditions and were less vulnerable to certain diseases, especially in the early 20^th^ century. When the analyzed older adults were children, public health measures were more effective in urban areas than in rural areas. This contributed to the lower spread of communicable diseases. The fact that these old people had been less exposed to diseases and epidemics may have contributed to their lengthier survival, as is true regarding the better health conditions.

Excluding the variables that represent the conditions from early life had almost no effect on the socioeconomic and lifestyle variables on mortality (the F value was slightly different: going from 22.01 to 23.02). A different situation was observed when the childhood conditions in studies that focus on the health of older adults were incorporated. Using data from the 2000 study SABE, Campos^4^ evaluated the associations among the socioeconomic conditions throughout the lives of older adults and three different approaches to health evaluation. This author concluded that older adults who had the most vulnerable conditions during childhood tend to have the highest number of chronic diseases, the greatest functional limitations and the most negative view of their own health. Santos et al^b^ showed that where older adults lived during childhood (rural or urban) is related to a perception of health among older adults. Similar results were observed in the Chinese population aged 80 years and over.^27^

These results have some limitations. The first limitation is related to the nature of the research. The SABE study does not include institutionalized individuals and can therefore underestimate some indicators that are related to health, since the prevalence of disability tends to be greater in this subgroup of the population.^5^ Nevertheless, Lima-Costa and Barreto^14^ stress that this bias is more significant among older adults of more advanced age, since the probability of institutionalization tends to grow with age and in communities with a higher degree of institutionalization.

The database is subject to selection effects. The information only relates to living older adults, meaning that any associations found are selectively bias. In addition, the information from the SABE study is obtained by means self-reporting, thusly, the answers could be influenced by cultural factors, memory, education and emotional and psychological issues. Having quality, accurate information can mitigate or strengthen the effects of the estimated parameters in the models, especially when variables referring to older adults’ first years of life are analyzed.

Any older adult who submitted scores of 12 points or less during their cognitive assessment required another informant to help them respond to the interview questions. Despite there being doubt regarding the validity of the information provided by the respondents, there have not been many studies to address this problem.^9^ Lima-Costa et al,^19^ while analyzing data from the 1998 and 2003 *Pesquisa Nacional por Amostra de Domicílios *(PNAD – National Household Sample Survey) and a cohort study of older adults residents in Bambui, Minas Gerais (Bambui Project), Southeastern Brazil, the authors concluded that it is not possible to determine *a priori* whether the answers provided by third parties affect the distribution of the variables. This will depend on the criteria adopted to define the participation of a respondent replacement. The criterion adopted in the SABE study is considered to be a valid and reliable instrument because, in addition to analyzing the ability for calculation and guidance, it also includes the evaluation of short-term memory loss. Only 9.3% of the older adults had help to answer some of their questions. Even if all the older adults had responded to the questionnaire unaided, the results would not be expected to present any significant changes.

All the variables regarding health and socioeconomic conditions during childhood referred to the first 15 years of life. This information is valuable, not extensively available and rarely unedited when compared to other databases, which makes it possible for the direct association to be evaluated along with the current outcomes.^b^ Meanwhile, as is true for any retrospective information, memory errors and missing data exist on those who, in similar circumstances of life, were not interviewed because they died beforehand. In addition, the measures available regarding conditions during childhood are limited and may not accurately measure the socioeconomic status and health during their first years of life. Information that is more commonly used in the studies relating to mortality and conditions during childhood, such as occupation or parent educational level, individual height and weight at birth,^8^ were not collected and therefore could not be used in this study. If these variables had been considered in these analyses, information regarding conditions during childhood could have remained statistically significant even after including the characteristics acquired by the individuals during adulthood.^8^

The time when the older adults came to the city of Sao Paulo (whether as a child or an adult) should be considered. The various regions of Brazil are characterized by different levels of urbanization and economic development and it is this aspect that can have an influence on lifestyle and other factors related to mortality. In addition, even if the analyzed subjects were located in the same geographic space, they belonged to different birth cohorts and experienced distinct political and social contexts.

These results are restricted to the older adults living in Sao Paulo in 2000. Generalizations must be performed carefully. Similar studies in other parts of Brazil are required to better understand how these determinants of mortality behave in different Brazilian regions. Doing this would make identifying factors that deserve attention possible and may contribute to increasing the survival rate of that analyzed population.

The SABE study was also performed in six other Latin American countries. Similar investigations may clarify understanding regarding the mechanisms related to mortality in older adults, since studies of this type are not greatly documented in developing countries.

## Footnotes

a Allison PD. Logistic regression using the SAS system: theory and application. Cary: SAS Institute; 1999.

b Santos JLF, Oliveira ML, Souza MR, Lebrão ML, Duarte YAO. O meio rural e a origem do idoso: a saúde e a morte na cidade (resultados do Estudo SABE 2000-2006). In: Anais do 17 Encontro Nacional de Estudos Populacionais; 20-24 set 2010; Caxambu, Brasil. Belo Horizonte (MG): ABEP; 2010.
