# Supplementary material for: Past and present: conditions of life during childhood and mortality of older adults
Source: Rev Saude Publica. 2016 Jan 12;49:93. doi: 10.1590/S0034-8910.2015049005555 (PMC4716652; doi:10.1590/S0034-8910.2015049005555)
Supplement: Translation

# Translation: Passado e presente: condições de vida na infância e mortalidade de idosos

**Authors:** Marília Miranda Forte Gomes, Cássio Maldonado Turra, Moema Gonçalves Bueno Fígoli, Yeda A O Duarte, Maria Lúcia Lebrão

**Affiliations:** I Faculdade do Gama. Universidade de Brasília. Brasília, DF, Brasil; II Centro de Desenvolvimento e Planejamento Regional. Faculdade de Ciências Econômicas. Universidade Federal de Minas Gerais. Belo Horizonte, MG, Brasil; III Departamento de Enfermagem Médico-Cirúrgica. Escola de Enfermagem. Universidade de São Paulo. São Paulo, SP, Brasil; IV Departamento de Epidemiologia. Faculdade de Saúde Pública. Universidade de São Paulo. São Paulo, SP, Brasil

**Keywords:** Idoso, Mortalidade, Desenvolvimento Infantil, Condições Sociais, Fatores Socioeconômicos

## Abstract

**OBJETIVO:**

: Analisar se as condições socioeconômicas e de saúde na infância estão associadas à mortalidade em idosos.

**MÉTODOS:**

: Utilizaram-se os dados do Estudo SABE (Saúde, Bem-estar e Envelhecimento) realizado em 2000 e 2006. A amostra foi composta por 2004 idosos, sendo 1.355 idosos sobreviventes e 649 óbitos. Modelos de Regressão de Poisson foram estimados, levando-se em consideração a variação do tempo de risco. Foram avaliadas características demográficas, condições no início da vida do idoso e condições socioeconômicas e de estilo de vida adquiridos na fase adulta.

**RESULTADOS:**

: Situação de residência (rural ou urbano) quando criança associou-se à mortalidade nas idades avançadas. No entanto, essa variável perdeu significância quando variáveis que representam características adquiridas na fase adulta foram adicionadas ao modelo.

**CONCLUSÕES:**

: Mesmo que as informações disponíveis sobre condições na infância sejam limitadas e possam não medir com precisão o *status* socioeconômico e de saúde nos primeiros anos de vida, os achados sugerem que melhorias nas condições ambientais das crianças e a criação de oportunidades no início da vida adulta podem contribuir para maior sobrevivência nas idades mais avançadas.

## INTRODUÇÃO

O termo ‘condições de vida na infância ou início da vida’ é amplamente utilizado pela literatura.^22^ Refere-se a um conjunto heterogêneo de fatores constituído pelo estado nutricional, do útero à infância, crescimento e desenvolvimento fisiológico nos primeiros anos de vida, exposição e contração de doenças infecciosas e parasitárias, experiência com ambientes estressantes e, mais genericamente, pelas experiências associadas às condições socioeconômicas da família na infância.

A partir das últimas décadas do século XX, têm sido investigado se indivíduos expostos a condições socioeconômicas e de saúde adversas quando crianças apresentam maior ou menor sobrevivência na fase adulta ou nas idades mais avançadas. Segundo Preston, Hill e Drevenstedt,^25^ existem pelo menos quatro mecanismos que relacionam condições na infância e mortalidade adulta. Esses mecanismos podem atuar diretamente, representando a influência fisiológica das condições de saúde na infância e na mortalidade de adultos e, indiretamente, quando associados a variáveis não fisiológicas.

Ao atuar diretamente, os mecanismos poderiam produzir associação positiva ou negativa nos riscos de mortalidade na fase adulta. Na associação positiva – *scarring* ou efeito colateral – condições adversas e doenças adquiridas na infância podem diminuir a probabilidade de sobrevivência nas idades mais avançadas. Baixo peso ao nascer e subnutrição nos primeiros anos de vida podem resultar em prejuízos fisiológicos. Além de prejudicar o desenvolvimento integral de órgãos funcionais, a má nutrição na infância pode tornar os indivíduos adultos mais vulneráveis a doenças não transmissíveis, especialmente doenças cardiovasculares e diabetes, contribuindo para maior risco de morte.^1^ ^,^ ^6^ ^,^ ^7^ ^,^ ^10^

A relação negativa associa-se à imunidade adquirida pelo indivíduo ao longo da vida. Indivíduos sobreviventes, frequentemente expostos a certas doenças na infância, como gripe, asma e varíola, podem apresentar risco de morte menor na fase adulta que aqueles que foram expostos a ambientes mais saudáveis. A exposição sucessiva a enfermidades pode aumentar a função autoimune e reduzir o risco de morte.^9^ ^,^ ^22^

Quanto à direção da relação indireta, os mecanismos que apresentam associações positivas podem estar correlacionados às condições do meio em que os indivíduos vivem. Aqueles que nasceram sob circunstâncias favoráveis (pai ou mãe pertencente a classes ocupacionais de melhor *status*, alimentação saudável disponível, moradia em áreas menos expostas a epidemias e acesso à educação e a serviços de saúde de qualidade) tendem a reter as vantagens ao longo da vida e são mais suscetíveis a ganhos no número médio de anos vividos na fase adulta e na velhice. A relação positiva indireta indica que os indivíduos que experimentaram condições adversas relacionadas ao meio, na infância, tendem a apresentar maior risco de mortalidade nas idades adultas.^9^ ^,^ ^23^ ^,^ ^27^ Por outro lado, as associações negativas relacionam-se à seletividade da mortalidade, ou seja, independentemente das condições de vida, os indivíduos mais frágeis morrem quando jovens e somente os mais fortes sobrevivem às idades mais avançadas.^25^ ^,^ ^27^

Boa parte da população idosa do Brasil teve a infância marcada por condições socioeconômicas e de saúde precárias. Se no final do século XIX e início do XX, a situação da estrutura urbana e social das cidades era de pobreza e de más condições de vida, atualmente as cidades são urbanizadas e oferecem maior quantidade de serviços e informação que contribuem para melhores condições de saúde e de estilo de vida.^21^ A longevidade média da população mundial, mais especificamente a brasileira, cresceu muito durante o século XX. Esses trabalhos^13^ ^,^ ^20^ ^,^ ^24^ ^,^ ^25^ são cada vez mais importantes para compreender os fatores associados à mortalidade de diferentes segmentos populacionais.^9^ A investigação dos determinantes associados às condições na infância e à sobrevivência nas idades avançadas contribuem não só para temas como a mortalidade adulta e políticas públicas focalizadas à população mais velha, como também para o planejamento e a implantação de políticas sociais focadas nos primeiros anos de vida. Isso pode garantir maiores ganhos de sobrevivência para o indivíduo quando adulto.^27^

Assim, este artigo teve como objetivo analisar se as condições socioeconômicas e de saúde na infância estão associadas à mortalidade em idosos.

## MÉTODOS

O presente artigo utilizou dados do Estudo SABE, Saúde, Bem-estar e Envelhecimento, realizado nos anos 2000 e 2006 no município de São Paulo, com base em amostra representativa da população urbana de 60 anos ou mais.^26^ A amostra do SABE foi constituída em duas fases distintas. Na primeira, usando o método de amostragem por conglomerados, foram visitados 90 domicílios em 72 setores censitários. Foram extraídos de um cadastro permanente, disponível no Departamento de Epidemiologia da Faculdade de Saúde Pública da Universidade de São Paulo, constituído a partir do cadastro da Pesquisa Nacional por Amostra de Domicílios do Instituto Brasileiro de Geografia e Estatística (IBGE). Os indivíduos elegíveis (idosos, com 60 anos ou mais, não institucionalizados e residentes nas áreas urbanas do município de São Paulo). foram identificados e convidados a participar das entrevistas. Na primeira fase da pesquisa,^16^ ^,^ ^17^ 1.568 idosos foram entrevistados. Na segunda fase, a amostra foi acrescida de 575 idosos com 75 anos ou mais, residentes em moradias próximas aos setores selecionados ou, no máximo, dentro dos limites dos distritos aos quais pertenciam os setores sorteados, totalizando 2.143 idosos. O objetivo foi compensar o efeito da mortalidade e completar o número desejado de entrevistas nesse grupo etário.

Entre os 2.143 idosos (100%) não institucionalizados e residentes nas áreas urbanas do Município de São Paulo, participantes da primeira fase do estudo em 2000, 1.115 (52,0%) foram novamente entrevistados entre julho de 2006 e dezembro de 2007. A diferença entre o número de entrevistados em 2000 e 2006 (1.028 idosos) foi composta por óbitos para todas as causas de morte (30,0%), institucionalizações (1,0%), mudanças (2,0%), idosos não encontrados (7,0%) e recusas (8,0%). A condição de sobrevivência de cada idoso foi determinada ao final do período de observação (Figura). A amostra analisada foi de 2004 idosos, sendo 1.355 sobreviventes e 649 óbitos. A diferença de 139 idosos excluídos foi composta por indivíduos entrevistados em 2000, mas para os quais não foi possível determinar sua condição de sobrevivência em 2006. Esta exclusão não alterou o padrão de distribuição das variáveis utilizadas nos modelos de análise, segundo resultados de testes Qui-quadrado (p > 0,10).

**Figura:** Estado final considerado na amostra analisada. São Paulo, SP, 2000 a 2006.

Fonte: Estudo SABE 2000-2006.

Foram selecionadas 15 variáveis para analisar a relação entre mortalidade e condições nos primeiros anos de vida. As variáveis foram organizadas em quatro grupos (Tabela 1): características básicas demográficas; condições no início da vida; condições sociais e econômicas adquiridas na fase adulta; e estilo de vida na fase adulta. As variáveis explicativas referem-se às informações declaradas em 2000, ano da primeira entrevista da SABE (linha de base).

**Tabela 1:** Variáveis explicativas utilizadas para analisar a relação entre mortalidade e condições nos primeiros anos de vida. São Paulo, SP, 2000 a 2006.

Variável	Descrição*	Categorização
Nacionalidade	O(A) sr.(a) nasceu no Brasil? (A2)	Sim
		Não
Grupos de idade	Ano de nascimento (A1a)	60 a 69 anos (sexagenários)
	Ano do óbito (dtobito)	70 a 79 anos (septuagenários)
	Ano da primeira entrevista (entrev_ANO1)	≥ 80 anos
	Ano da segunda entrevista (entrev_ANO2)	
Sexo	Sexo do entrevistado (C18)	Homem
		Mulher
Passou fome?	Durante os primeiros 15 anos da sua vida, o(a) sr.(a) diria que houve algum tempo em que não comeu o suficiente ou passou fome? (C30)	Sim
		Não
Situação econômica da família	Como o(a) sr.(a) descreveria a situação econômica de sua família durante a maior parte dos primeiros 15 anos de sua vida? (C26)	Boa
		Regular/Ruim
Viveu no campo?	Desde que o sr.(a) nasceu até os 15 anos, viveu no campo por 5 anos ou mais? (A4b)	Sim
		Não
Autoavaliação da saúde	Durante a maior parte dos primeiros 15 anos da sua vida, o(a) sr.(a) descreveria sua saúde naquela época como: (C27)	Excelente/Boa
		Ruim
Acamado	Durante os primeiros 15 anos da sua vida ficou na cama por um mês ou mais devido a algum problema de saúde? (C29)	Sim
		Não
Nível de escolaridade	Número de anos de estudo (A5a, A5b, A6)	Nenhum
		1 a 7 anos
		≥ 8 anos
Tem casa própria?	Esta casa é: (J2)	Sim
		Não
Estado nutricional	Com relação a seu estado nutricional o(a) sr.(a) se considera bem nutrido? (C22i)	Bem nutrido
		Não está bem nutrido
Estado marital	Situação conjugal (A13a, A15, A16)	Casado
		Divorciado/Separado
		Viúvo
		Solteiro
Hábito de fumar	O(A) sr.(a) tem ou teve o hábito de fumar? (C24)	Fuma atualmente
		Já fumou mais não fuma mais
		Nunca fumou
Uso de álcool	Nos últimos três meses, em média, quantos dias por semana tomou bebida alcoólica? (C23)	Não faz uso
	Nos últimos três meses, nos dias em que tomou bebida alcoólica, foram quantos copos, em média, por dia? (C23a)	Faz uso
Prática de atividade física	Nos últimos 12 meses, tem feito exercícios ou realizado atividades físicas vigorosas regularmente, 3 vezes por semana? (C25a)	Sim
		Não

Fonte: Elaboração própria.

* Os códigos em parênteses na descrição das variáveis se referem à identificação da variável no banco de dados do Estudo SABE.

Dos 2.004 idosos analisados, 148 apresentaram valores faltantes em algumas das variáveis explicativas. Testes Qui-quadrado foram realizados para examinar a aleatoriedade dessas não respostas e possível perda de poder estatístico ao estimar os modelos multivariados. A exclusão desses valores em branco não alterou o padrão de distribuição das variáveis. Assim, a amostra final foi composta de 1.856 idosos, dos quais 1.276 sobreviventes e 580 óbitos.

Modelos de Regressão de Poisson foram utilizados para analisar a relação entre o número de mortes de 2000 a 2006 e as variáveis explicativas selecionadas.^15^ ^,^ ^a^ Investigou-se isoladamente a relação entre cada variável explicativa e a variável resposta, representada pela condição de sobrevivência dos idosos (sobrevivente ou óbito). As variáveis explicativas que apresentaram p < 0,20 foram consideradas elegíveis para compor os modelos de regressão de Poisson múltiplos. Esse procedimento foi adotado para tornar mais parcimoniosa a escolha das variáveis explicativas a partir da literatura internacional.^14^ ^,^ ^23^ As variáveis excluídas da lista inicial referiam-se às condições no início da vida: situação econômica da família, autoavaliação da saúde e se ficou na cama por um mês ou mais devido a algum problema de saúde. Quatro modelos foram estimados para examinar os efeitos das condições de infância sobre a mortalidade.

Seguindo Gomes et al,^11^ a fim de detectar uma possível existência de multicolinearidade (dependência linear perfeita ou aproximada entre pelo menos duas variáveis explicativas),^11^ ^,^ ^12^ analisou-se o *Variance Inflation Factor* (VIF) em cada um dos modelos. O VIF é indicativo de problemas de multicolinearidade se apresentar valores maiores que 10. O valor médio dessa medida não foi superior a 1,5 e nenhuma variável apresentou VIF maior que 2,7 em todos os modelos multivariados estimados.

Para as estimativas dos modelos apresentados foram considerados os pesos amostrais e o plano amostral delineado para o Estudo SABE.^26^ Para tanto, utilizaram-se rotinas específicas para o tratamento de dados provenientes de amostras complexas^12^ disponíveis no programa Stata, versão 9.0. Descrição mais detalhada de como os pesos amostrais foram calculados encontra-se em Silva.^26^

## RESULTADOS

O nível de mortalidade foi significativamente maior para idosos do sexo masculino e para aqueles dos grupos de idade mais avançados. Quanto maior o nível de escolaridade e o *status* socioeconômico (ter casa própria), maior foi a sobrevivência (Tabela 2). Nível de mortalidade estatisticamente mais alto foi observado para idosos que autoavaliaram o seu estado nutricional como ruim, para os viúvos, fumantes, os que não praticavam exercícios regularmente e os que faziam uso regular de bebida alcoólica. O nível de mortalidade foi 26,6% menor para idosos que viveram pelo menos cinco anos em área urbana até os 15 anos relativamente aos que viveram em áreas rurais.

**Tabela 2:** Distribuição relativa e análise univariada, por condição de sobrevivência, segundo variáveis explicativas selecionadas para os modelos múltiplos. São Paulo, SP, 2000 e 2006. (N = 1.856)

Variável	Frequência relativa		Análise univariada		

	Sobrevivente	Óbito	Coeficiente	RTM	p
Nacionalidade					
Brasileiro	90,6	85,8	–		
Estrangeiro	9,4	14,2	0,293	1,340	0,120
Grupos de idade					
Sexagenário	45,1	20,3	–		
Septuagenário	39,3	36,9	0,558	1,747	< 0,001
≥ 80 anos	15,7	42,7	1,415	4,116	< 0,001
Sexo					
Homem	37,1	52,0	–		
Mulher	62,9	48,0	-0,451	0,637	< 0,001
Passou fome?					
Sim	19,7	16,1	–		
Não	80,3	83,9	0,176	1,192	0,131
Situação econômica da família					
Boa	30,7	32,0	–		
Regular/Ruim	69,3	68,0	-0,766	0,465	0,502
Viveu no campo?					
Sim	60,8	70,1	–		
Não	39,2	29,9	-0,310	0,734	0,018
Autoavaliação da saúde					
Excelente/Muito boa/Boa	93,7	96,1	–		
Regular/Ruim	6,3	3,9	-0,283	0,753	0,260
Acamado					
Sim	7,8	7,7	–		
Não	92,2	92,3	-0,007	0,993	0,970
Nível de escolaridade					
Nenhuma	20,0	33,0	–		
1 a 7 anos	63,4	56,2	-0,465	0,628	< 0,001
≥ 8 anos	16,6	10,8	-0,793	0,453	< 0,001
Tem casa própria?					
Sim	83,1	77,8	–		
Não	16,9	22,2	0,345	1,412	0,008
Estado nutricional					
Bem nutrido	94,4	91,8	–		
Não está bem nutrido	5,6	8,2	0,332	1,394	0,044
Estado marital					
Casado	56,0	50,1	–		
Divorciado/Separado	7,6	4,8	-0,121	0,886	0,603
Viúvo	32,1	40,9	0,273	1,314	0,039
Solteiro	4,3	4,3	0,187	1,206	0,371
Hábito de fumar					
Fuma atualmente	11,8	16,9	–		
Já fumou, mas não fuma mais	30,5	35,6	-0,379	0,685	0,010
Nunca fumou	57,8	47,5	-0,655	0,520	< 0,001
Uso de álcool					
Não faz uso	67,7	76,2	–		
Faz uso	32,3	23,8	-0,298	0,743	0,031
Prática de atividade física					
Sim	29,2	12,6	–		
Não	70,8	87,4	0,814	2,256	< 0,001

Fonte: Estudo SABE 2000-2006.

RTM: Razão entre as taxas de mortalidade.

O modelo 1, múltiplo, incluiu as informações que representam condições no início da vida e as características demográficas básicas. As variáveis ‘nacionalidade’ (p = 0,978) e ‘passar fome na infância’ (p = 0,302) não se mostraram associadas com a mortalidade. Os idosos que viveram em áreas urbanas no início da vida apresentaram taxas de mortalidade aproximadamente 20,0% menor do que aqueles que viveram em áreas rurais (p < 0,10) (Tabela 3).

**Tabela 3:** Resultados dos modelos multivariados estimados para análise da relação entre condições na infância e mortalidade de idosos. São Paulo, SP, 2000 e 2006.

Variável	Modelo 1			Modelo 2			Modelo 3			Modelo 4		
	Coeficiente	RTM	p	Coeficiente	RTM	p	Coeficiente	RTM	p	Coeficiente	RTM	p
Características individuais												
Nacionalidade												
Brasileiro	–	1,000		–	1,000		–	1,000		–	1,000	
Estrangeiro	0,005	1,005	0,978	0,059	1,060	0,751	0,133	1,142	0,481	0,136	1,146	0,459
Grupos de idade												
Sexagenário	–	1,000		–	1,000		–	1,000		–	1,000	
Septuagenário	0,576	1,779	< 0,001^a^	0,527	1,694	0,001^a^	0,551	1,735	< 0,001^a^	0,564	1,757	< 0,001^a^
≥ 80 anos	1,468	4,338	< 0,001^a^	1,346	3,842	< 0,001^a^	1,330	3,781	< 0,001^a^	1,347	3,845	< 0,001^a^
Sexo												
Homem	–	1,000		–	1,000		–	1,000		–	1,000	
Mulher	-0,535	0,586	< 0,001^a^	-0,680	0,507	< 0,001^a^	-0,641	0,527	< 0,001^a^	-0,640	0,528	< 0,001^a^
Condições no início da vida												
Passou fome?												
Sim	–	1,000		–	1,000		–	1,000				
Não	0,127	1,135	0,302	0,194	1,215	0,134	0,220	1,246	0,110			
Viveu no campo?												
Sim	–	1,000		–	1,000		–	1,000				
Não	-0,226	0,798	0,066^b^	-0,131	0,877	0,274	-0,097	0,908	0,414			
Fase adulta												
Nível de escolaridade												
Nenhuma				–	1,000		–	1,000		–	1,000	
1 a 7 anos				-0,254	0,775	0,015^c^	-0,135	0,874	0,195	-0,151	0,860	0,129
≥ 8 anos				-0,534	0,586	0,012^c^	-0,347	0,707	0,100	-0,366	0,693	0,082^b^
Tem casa própria?												
Sim				–	1,000		–	1,000		–	1,000	
Não				0,331	1,392	0,009^a^	0,299	1,349	0,014^c^	0,290	1,336	0,019^c^
Estado nutricional												
Bem nutrido				–	1,000		–	1,000		–	1,000	
Não está bem nutrido				0,397	1,488	0,018^c^	0,233	1,263	0,134	0,193	1,212	0,221
Estado marital												
Casado				–	1,000		–	1,000		–	1,000	
Divorciado/separado				0,033	1,034	0,888	-0,001	0,999	0,997	-0,013	0,987	0,949
Viúvo				0,175	1,191	0,196	0,167	1,182	0,216	0,158	1,172	0,237
Solteiro				0,255	1,290	0,202	0,251	1,285	0,162	0,226	1,253	0,191
Hábito de fumar												
Fuma atualmente							–	1,000		–	1,000	
Já fumou, mas não fuma mais							-0,469	0,626	0,002^a^	-0,484	0,616	0,000^a^
Nunca fumou							-0,634	0,531	< 0,001^a^	-0,636	0,529	< 0,001^a^
Uso de álcool												
Não faz uso							–	1,000		–	1,000	
Faz uso							-0,276	0,759	0,035^c^	-0,273	0,761	0,035^c^
Prática de atividade física												
Sim							–	1,000		–	1,000	
Não							0,611	1,842	< 0,001^a^	0,609	1,838	< 0,001^a^
Tamanho da amostra	1.856			1.856								
Constante	-3,56		< 0,001^a^	-3,47		< 0,001^a^	-3,54		< 0,001^a^	-3,39		< 0,001^a^
Valor de F	26,81			23,02								
Valor de p	< 0,001			< 0,001								

Nota: O primeiro modelo inclui as características demográficas básicas dos indivíduos e as variáveis que representam suas condições de vida na infância. Os Modelos 2 e 3 têm como objetivo medir o papel de variáveis intervenientes que representam características adquiridas na vida adulta: o modelo 2 adiciona as condições sociais e econômicas e o terceiro modelo incorpora as variáveis que representam estilo vida. Estimou-se um quarto modelo que exclui as variáveis sobre condições no início da vida. Seu objetivo é examinar em que medida a omissão das condições na infância afeta o poder explicativo dos modelos tradicionais de mortalidade adulta que levam em conta apenas as características adquiridas nas fases mais tardias do ciclo de vida.

Fonte: Estudo SABE 2000-2006.

RTM: Razão entre as taxas de mortalidade

^a^ p < 0,01.

^b ^p < 0,10.

^c^ p < 0,05.

A variável ‘viveu no campo’ perdeu significância estatística (p > 0,10) no modelo 2, que incluiu as condições socioeconômicas adquiridas nas idades adultas. Assim, idosos com mais anos de estudos, que possuíam casa própria ou que avaliaram o seu estado nutricional positivamente apresentaram maior sobrevivência, mesmo na presença dos controles para as condições na infância (p < 0,05). O modelo 3 adicionou variáveis sobre o estilo de vida dos idosos. As variáveis acerca das condições na infância permaneceram sem significância estatística (p > 0,10). Efeitos da educação passaram a ser marginalmente significativos (p = 0,10) e o estado nutricional deixou de ser significativo (p > 0,10).

No modelo 4, ao excluir as variáveis que representam as condições do início da vida, os efeitos das variáveis socioeconômicas e de estilo de vida sobre a mortalidade quase não foram alterados (todos significativos ao nível de 5%). Escolaridade, que no modelo 3 era marginalmente significativa ao nível de 10,0%, passou para p = 0,082 (Tabela 3)

## DISCUSSÃO

Na primeira análise múltipla, quando consideradas apenas as variáveis demográficas e de condições na infância, houve relação negativa e significativa entre mortalidade e não ter vivido na área rural até completar 15 anos de idade (p < 0,10), ou seja, idosos que residiram em localidades urbanas quando crianças apresentaram menor prevalência de óbitos do que aqueles que viveram no campo. No entanto, depois de controlar separadamente as condições socioeconômicas e de estilo de vida corrente, o efeito de ter vivido em localidades rurais nos primeiros anos de vida sobre o risco de mortalidade dos idosos deixou de ser estatisticamente significativo. Ao excluir do modelo múltiplo as variáveis que representam as condições do início da vida, os efeitos das variáveis socioeconômicas e de estilo de vida sobre a mortalidade quase não foram alterados.

Os resultados deste estudo confirmam os de outros países. Condições na infância deixarem de ser estatisticamente significativas quando as socioeconômicas e de estilo de vida corrente são acrescidas ao modelo, conforme relatado: por Bobak et al,^3^ em estudo sobre determinantes de mortalidade da população adulta e idosa residente na Rússia; por Hayward e Gorman,^13^ para a população idosa americana; por Beebe-Dimmer et al,^2^ entre mulheres adultas e idosas residentes na Califórnia; e por Yi, Gu e Land,^27^ ao analisar a população idosa residente na China (p < 0,10). Os autores concluem que as condições adquiridas na vida adulta agem, de maneira significativa, como fatores intermediários na relação entre características socioeconômicas e de saúde nos primeiros anos de vida e mortalidade nas idades mais avançadas.

Resultados na literatura são controversos quanto aos idosos residentes em localidades rurais quando crianças apresentarem maior mortalidade. Diferentemente do que foi observado por Hayward e Gorman^13^ e por Preston, Hill e Drevenstedt,^25^ no presente estudo e no realizado por Yi, Gu e Land,^27^ os idosos que viveram em áreas urbanas quando crianças apresentaram menor risco de mortalidade em comparação com aqueles que moravam em localidades rurais. Segundo Preston et al,^25^ isso pode ser explicado pelo fato das localidades urbanas apresentarem melhores condições socioeconômicas, ambientais e de menos vulnerabilidade para certas doenças, especialmente no início do século XX. Quando os idosos analisados eram crianças, medidas de saúde pública foram mais eficazes nas áreas urbanas do que nas rurais. Isso contribuiu para menor propagação de doenças transmissíveis. O fato de esses idosos terem sido menos expostos a doenças e epidemias pode ter colaborado para que sobrevivessem durante mais tempo, inclusive com melhores condições de saúde.

Excluir as variáveis que representam as condições do início da vida quase não alterou os efeitos das variáveis socioeconômicas e de estilo de vida sobre a mortalidade (o valor de F pouco se modificou: passou de 22,01 para 23,02). Situação diferente foi observada ao incorporar as condições na infância em estudos que têm como foco a saúde do idoso. Utilizando dados do Estudo SABE 2000, Campos^4^ avaliou as associações entre as condições socioeconômicas ao longo da vida dos idosos e três diferentes enfoques de avaliação da saúde. O autor concluiu que os idosos com condições mais vulneráveis na infância tendem a apresentar maior número de doenças crônicas, possuir maiores limitações funcionais e avaliar negativamente suas condições de saúde. Santos et al^b^ mostraram que o local onde o idoso viveu na infância (zona rural ou urbana) está relacionado com a percepção de saúde entre os idosos. Resultados similares foram observados para a população chinesa com 80 anos e mais.^27^

Os resultados apresentam algumas limitações. A primeira está relacionada com a natureza da pesquisa. O Estudo SABE não inclui indivíduos institucionalizados e isso pode subestimar alguns indicadores relacionados às condições de saúde, já que a prevalência de incapacidades tende a ser maior nesse subgrupo da população.^5^ Lima-Costa e Barreto,^14^ no entanto, salientam que esse viés é mais acentuado entre os idosos mais velhos, já que a probabilidade de institucionalização tende a crescer com a idade e em comunidades com maior grau de institucionalização.

A base de dados está sujeita a efeitos de seleção. As informações referem-se apenas aos idosos sobreviventes, fazendo com que as associações encontradas tenham viés de seletividade. Além disso, as informações do Estudo SABE são fornecidas por meio de autorrelato e, assim, as respostas podem ser influenciadas por fatores culturais, de memória, pela escolaridade e por questões emocionais e psicológicas. A qualidade da precisão das informações pode atenuar ou fortalecer os efeitos dos parâmetros estimados nos modelos, especialmente quando são analisadas variáveis referentes aos primeiros anos de vida dos idosos.

Foi necessário que outro informante auxiliasse o idoso a responder a entrevista para aqueles que apresentaram pontuação igual ou inferior a 12 pontos no teste de avaliação cognitiva. Embora se questione a validade das informações fornecidas por respondentes substitutos, poucos são os estudos que abordaram de fato o problema.^9^ Lima-Costa et al,^19^ ao analisarem os dados da Pesquisa Nacional por Amostra de Domicílios (PNAD) de 1998 e 2003 e de uma coorte de idosos residentes em Bambuí, MG (Projeto Bambuí), concluíram que não é possível determinar *apriori* se as respostas fornecidas por terceiros afetam a distribuição das variáveis. Isso dependerá dos critérios adotados para definir a participação de um respondente substituto. O critério adotado no Estudo SABE é considerado instrumento válido e confiável, pois, além de analisar a habilidade de cálculo e de orientação, inclui também a avaliação da perda de memória recente. Apenas 9,3% dos idosos tiveram ajuda para responder algumas perguntas. Espera-se que, mesmo que todos os idosos analisados tivessem respondido ao questionário sem nenhum auxílio, os resultados apresentados não apresentariam alterações significativas.

Todas as variáveis sobre condições de saúde e socioeconômica na infância se referiram aos primeiros 15 anos de idade. Essas informações são valiosas, pouco disponibilizadas ou até inéditas quando comparadas a outras bases de dados, permitindo que seja avaliada associação direta com os desfechos atuais.^b^ Entretanto, como em qualquer informação retrospectiva, está sujeita a erros de memória e não se dispõe dos dados referentes àqueles que, em idênticas circunstâncias de vida, não chegaram a ser entrevistados por terem falecido antes. Além disso, as medidas disponíveis sobre condições na infância são limitadas e podem não medir com precisão o *status* socioeconômico e de saúde nos primeiros anos de vida. Informações mais comumente utilizadas nos estudos que relacionam mortalidade e condições na infância, como ocupação ou escolaridade dos pais, estatura do indivíduo e peso ao nascer,^8^ não foram coletadas e, portanto, não puderam ser utilizadas neste estudo. Se essas variáveis tivessem sido consideradas nas análises apresentadas, as informações sobre condições na infância poderiam ter se mantido estatisticamente significativas, mesmo após a inclusão de características adquiridas na fase adulta.^8^

A época em que o idoso veio para o município de São Paulo (se quando criança ou se adulto) deve ser considerada. As regiões do Brasil são caracterizadas por níveis diferentes de desenvolvimento econômico e de urbanização e isso pode exercer influências no modo de vida e em outros fatores relacionados à mortalidade. Além disso, mesmo que os idosos analisados estejam localizados em um mesmo espaço geográfico, pertencem a coortes de nascimentos diferentes e experimentaram contextos políticos e sociais distintos.

Os resultados restringem-se aos idosos residentes em São Paulo em 2000. Generalizações devem ser realizadas de forma cautelosa. Estudos semelhantes em outras localidades do País são necessários para compreender melhor como os determinantes da mortalidade comportam-se nas diferentes regiões brasileiras. Isso permitiria a identificação de fatores que merecem atenção e que podem contribuir para maior sobrevivência do contingente populacional analisado.

O SABE também foi realizado em outros seis países da América Latina. Investigações similares podem clarear a compreensão dos mecanismos relacionados com a mortalidade dos idosos, já que estudos desse tipo são pouco documentados em países em desenvolvimento.

## Footnotes

a Allison PD. Logistic regression using the SAS system: theory and application. Cary: SAS Institute; 1999.

b Santos JLF, Oliveira ML, Souza MR, Lebrão ML, Duarte YAO. O meio rural e a origem do idoso: a saúde e a morte na cidade (resultados do Estudo SABE 2000-2006). In: Anais do 17º Encontro Nacional de Estudos Populacionais; 20-24 set 2010; Caxambu, Brasil. Belo Horizonte: ABEP; 2010.
